# Gene Therapy in Amyotrophic Lateral Sclerosis

**DOI:** 10.3390/cells11132066

**Published:** 2022-06-29

**Authors:** Ton Fang, Goun Je, Peter Pacut, Kiandokht Keyhanian, Jeff Gao, Mehdi Ghasemi

**Affiliations:** Department of Neurology, University of Massachusetts Chan Medical School, Worcester, MA 01655, USA; ton.fang@umassmemorial.org (T.F.); goun.je@umassmemorial.org (G.J.); peter.pacut@umassmemorial.org (P.P.); kiandokht.keyhanian@gmail.com (K.K.); jeff.gao2@umassmed.edu (J.G.)

**Keywords:** *C9orf72*, Cu/Zn superoxide dismutase (SOD1), TAR DNA binding protein 43 (TARDBP), fused in sarcoma (FUS), amyotrophic lateral sclerosis (ALS), gene therapy

## Abstract

Since the discovery of *Cu/Zn superoxide dismutase* (*SOD1*) gene mutation, in 1993, as the first genetic abnormality in amyotrophic lateral sclerosis (ALS), over 50 genes have been identified as either cause or modifier in ALS and ALS/frontotemporal dementia (FTD) spectrum disease. Mutations in *C9orf72*, *SOD1*, *TAR DNA binding protein 43* (*TARDBP*), and *fused in sarcoma* (*FUS*) genes are the four most common ones. During the last three decades, tremendous effort has been made worldwide to reveal biological pathways underlying the pathogenesis of these gene mutations in ALS/FTD. Accordingly, targeting etiologic genes (i.e., gene therapies) to suppress their toxic effects have been investigated widely. It includes four major strategies: (i) removal or inhibition of abnormal transcribed RNA using microRNA or antisense oligonucleotides (ASOs), (ii) degradation of abnormal mRNA using RNA interference (RNAi), (iii) decrease or inhibition of mutant proteins (e.g., using antibodies against misfolded proteins), and (iv) DNA genome editing with methods such as clustered regularly interspaced short palindromic repeats (CRISPR)/CRISPR-associated protein (CRISPR/Cas). The promising results of these studies have led to the application of some of these strategies into ALS clinical trials, especially for *C9orf72* and *SOD1*. In this paper, we will overview advances in gene therapy in ALS/FTD, focusing on *C9orf72*, *SOD1*, *TARDBP*, and *FUS* genes.

## 1. Introduction

Amyotrophic lateral sclerosis (ALS, also known as Lou Gehrig’s disease) and frontotemporal dementia (FTD) are two fatal neurodegenerative conditions that belong to a disease spectrum sharing clinical, genetic, and pathological findings. ALS affects upper motor neurons (UMNs) in the motor cortex and lower motor neurons (LMNs) in the brainstem and spinal cord [1]. The characteristic clinical manifestations include focal weakness spreading to all 4 limbs and bulbar muscles and hyperreflexia. The disease spectrum ranges from predominantly UMN (primary lateral sclerosis [PLS]) to predominantly LMN (progressive muscular atrophy [PMA]) disease. About 50% of patients with ALS may exert different degrees of cognitive dysfunction, and about 15% of patients with FTD may develop ALS phenotype [2,3]. Over 97% of patients with ALS and about 50% of those with FTD have histopathological findings of aggregation of TAR DNA-binding protein 43 (TDP-43) in both affected neurons and glial cells [4,5,6,7,8]. Autopsy findings have also revealed that degeneration of corticospinal tract and spinal/bulbar motor neurons are accompanied by activation of immune cells (i.e., microglia, astrocytes, and oligodendroglia) within the central nervous system (CNS) [9,10].

Although the vast majority of ALS cases are sporadic (sALS), about 10% of cases are familial (fALS) [3] with predominantly autosomal dominant and rarely X-linked or recessive inheritance [11,12]. In 1993, mutations in *cytosolic Cu/Zn superoxide dismutase* (*SOD1*) gene were identified as the first genetic abnormality in ALS [13]. Ever since, enormous efforts to identify mutated genes involved in ALS pathology have identified more than 50 genes and 120 genetic variants that increase the risk or modify the ALS phenotype [1,3,11,14]. Analysis of molecular pathways underlying these mutant ALS genes has robustly improved our knowledge about pathogenesis of both fALS and sALS, thereby providing new insights into potential targets for therapy. Overall, mutations in *SOD1*, chromosome 9 open reading frame 72 (*C9orf72*) [15,16,17], *TAR DNA binding protein 43* (*TARDBP or TDP-43*) [18], and *fused in sarcoma* (*FUS*) [19,20] genes have been found to be the four most common ones involved in over 70% of cases with fALS [3]. Accordingly, developing transgenic animal models and targeting the abnormal genes (i.e., gene therapy) has been investigated worldwide in order to translate these experimental gene therapies into the clinical setting. Although pre-clinical studies on different species may be challenging, as they may not truly represent the exact human phenotypes, the results of these studies have been promising and have led to the initiation of some of these strategies in ALS clinical trials. In this paper, we will overview advances in gene therapy in ALS and ALS/FTD focusing on *SOD1*, *C9orf72*, *TARDBP*, and *FUS* genes.

## 2. Strategies for Gene Therapy in ALS

**Figure 1:**
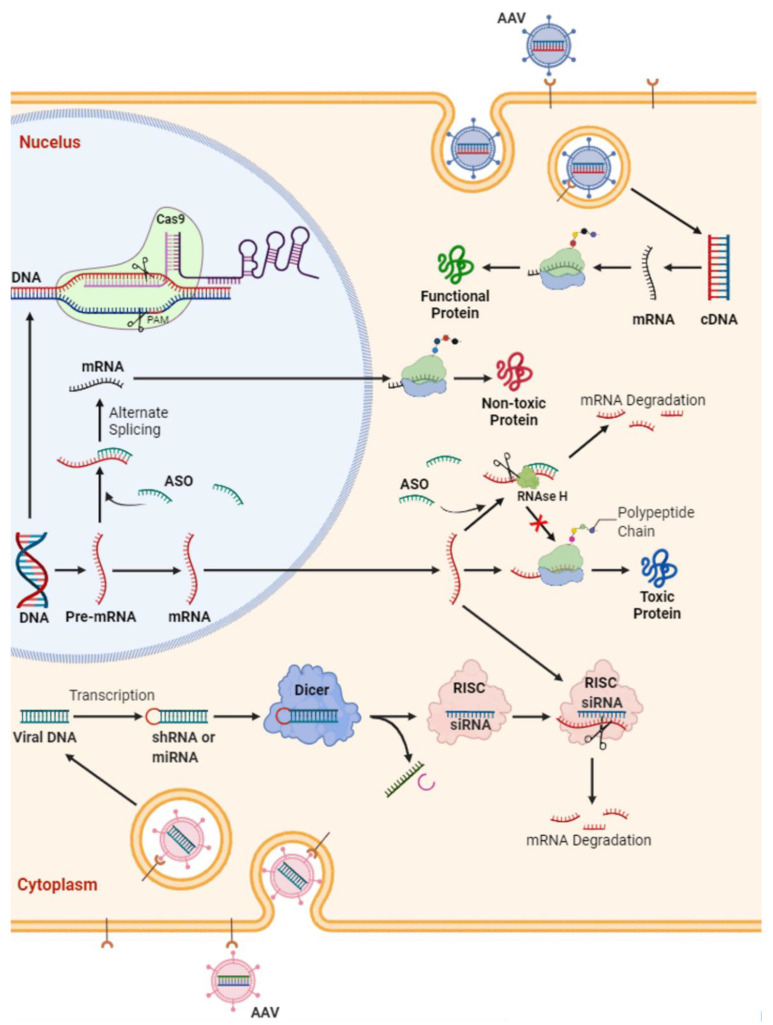
**Schematic representation of potential strategies in gene therapy for amyotrophic lateral sclerosis.** Antisense oligonucleotide (ASO) are short synthetic oligonucleotides (~20 nucleotides). They bind to the targeted mRNA and either (i) induce the mRNA degradation by endogenous RNase H or (ii) block the mRNA translation. This ultimately decreases the expression of certain proteins. In ALS, this strategy has been utilized to reduce the protein level of TDP-43, SOD1 of FUS protein level or to target *C9orf72* RNA foci. SiRNAs are double-stranded RNAs that can bind argonaute proteins as part of the RNA-induced silencing complex (RISC), which ultimately leads to the mRNA cleavage. Gene (i.e., either mRNA or cDNA) delivery through viruses (e.g., adeno-associated viral vectors [AAV]) is another option for functional replacement of a missing gene. This approach was utilized in spinal muscular atrophy but needs more investigation in ALS.

For a vast majority of genetic diseases, even single gene disorders, definite treatments are still lacking. In general, it takes several years of investigation to understand normal function of a pathogenic gene and molecular pathways underlying its pathogenesis. Even armed with this knowledge, developing techniques to target abnormal genes, especially in those with dominant traits, could take even longer. This is also true for ALS, in which 10–15% of cases are dominant, high-penetrance gene variants [14]. Overall, there are four approaches to suppressing the toxic effects of etiologic genes (Figure 1):

- MicroRNA or antisense oligonucleotides (ASOs; complementary DNA or RNA sequences designed to pair with the target sequence and activate RNA degradation) for ablation of the RNA transcribed from the gene: Administration of ASOs, which are synthetic nucleic acids targeting/altering mRNAs, have shown promising results in treatment of other neuromuscular disorders in children, such as spinal muscle atrophy (SMA) and Duchenne muscular dystrophy (DMD). This has completely altered the original disease trajectory, which has prompted FDA approval of two ASOs, nusinersin (Spinraza) and eteplirsen (Exondys51), for respective treatment of SMA type 1 and 2 and a subset of DMD; however, these have not been tested in adult-type SMA 3 and 4, and their utility in adult disease is not yet known. Overall, ASOs either selectively degrade mRNAs through recruitment of endonuclease RNase H or prevent the interaction of RNAs with RNA binding proteins (RBPs), thereby modulating their splicing/processing without degradation [21].
- Reduction in excess mutant protein (e.g., immune-mediated reduction).
- Interference with transcriptional process with the use of small molecules.
- Somatic-cell mutagenesis, a reverse mutation of the gene back to wild-type form.

Several reports have documented that the first three of these methods are feasible. The great advantage of the last approach is that correction of the mutant DNA eliminates downstream abnormalities and is, at least in theory, a one-time intervention.

## 3. *SOD1*

*SOD1* is a common gene target in ALS. First discovered in 1993 [22], *SOD1* mutations account for approximately 12–20 percent of hereditary ALS worldwide; in Asia, *SOD1* mutation is the most common cause of familial ALS [3,23]. The *SOD1* gene is located on chromosome 21 and encodes the enzyme Cu, Zn, superoxide dismutase. Normal function of SOD1 protein eliminates reactive oxygen species in cellular cytosol and mitochondria and thus is neuroprotective [24,25]. Therefore, mutation of this gene can lead to toxic gain or loss of function, which in turn disrupts normal cellular homeostasis. In ALS, neurodegeneration in *SOD1* mutation have been hypothesized to occur through a consortium of mechanisms such as oxidative stress, disruption of protein degradation, microglial inflammation, toxic protein aggregation, mitochondrial and oligodendrocytes dysfunction [14].

There have been over 170 different mutations described in *SOD1* [26]. Most of these are missense pathogenic variants that are transmitted in a dominant fashion. However, even with the same mutation, clinical presentation is unpredictable as there have been cases of phenotypic heterogeneity amongst patients that have inherited the same *SOD1* mutation [27,28]. Therefore, other factors such as epigenetics and environmental risk may be important for understanding disease expression in *SOD1* ALS. Certain *SOD1* mutations can also be predictors of ALS survival, such as the A5V mutation being associated with a mean 1 year survival [29], a particularly fast progressing subgroup of the population. Gene expression is complicated, including modulation from upstream promoter regions, epigenetic alterations, protein synthesis staging and cellular packaging, and with these complex steps, this widens the possibilities for *SOD1* and other gene targets for successful therapeutic approaches in ALS.

### 3.1. ASOs

ASOs are small molecules that mediate the degradation of both cytoplasmic mRNA and nuclear-retained RNA by targeting RNase H1-dependent degradation pathway, and in turn this reduces cellular protein synthesis [30]. In studies of other neurodegenerative diseases such as SMA, ASOs have already shown effectiveness at reducing all-cause mortality [31]. In the first trial using ASOs in ALS-targeted *SOD1*, researchers administered intrathecal injections of ASO into rats and rhesus monkeys and demonstrated across-the-board coverage in the CNS and found slowing of disease progression in ALS rat models [32]. In human trials, a phase I trial of intrathecal administration of ASOs targeting *SOD1* (ISIS 333611; different doses of 0.15, 0.50, 1.50, and 3.00 mg infused over 11.5 h; (Clinicaltrials.gov identifier: NCT01041222) [33,34] was found to be safe and well tolerated. A second-generation ASO tofersen, BIIB067 (IONIS-SOD1Rx), completed phase I/II trial (ClinicalTrials.gov identifier: NCT02623699) and found a dose dependent efficacy with highest dose 100 mg showing the largest of effects at reducing CSF SOD1 concentration, especially in fast progressors of disease [35] (Table 1). However, since participant numbers were low in the initial trial, BIIB067 was extended to a phase 3 clinical trial; however, the primary outcomes of measuring disease progression in ALS fast progressors at 28 weeks of treatment did not reach statistical significance [36]. A long-term phase 3 clinical trial for BIIB067 with follow-up for 7 years is currently in the planning stages (Clinicaltrials.gov identifier: NCT03070119). The issues with ASOs are that since the molecules work downstream to halt protein synthesis, if a successful molecule is found, it is likely that repeat doses would be required to counteract newly transcribed mRNA from the active gene in adult ALS patients.

### 3.2. RNAi

Another approach for targeting RNA/protein-related toxic gain of function in *SOD1* ALS pathology is using an RNA interference (RNAi) strategy. This differs from ASOs since RNAs are a double-stranded structure which, although more likely to survive delivery, requires stages of enzymatic processing before being active, compared to ASOs, which are single stranded and ready to directly bind to their target. During the RNAi process, RNAs destroy mRNAs in the cytoplasm through an RNA-induced silencing complex (RISC), thereby suppressing the expression of targeted genes [39]. The most common RNAi strategies consist of short interfering RNAs (siRNAs), short hairpin RNA (shRNAs), and artificial miRNAs. To mediate this, adeno-associated viral vectors (AAV) can be used to deliver RNAs into neurons in the CNS. In SOD1G93A mice models, AAV-mediated siRNA delivery led to a 39% survival benefit, with decreased efficacy based on the age of the mice, which would be expected since SOD1 homeostatic dysfunction would have already occurred in the advanced stage of disease. Several studies have shown efficacy of RNA with targets having lower expression of SOD-1 and saw outcomes of delay in disease onset and extension to survival [40,41,42].

These promising animal studies have led to a trial in two human subjects with familial ALS, using a single AAV-miR-SOD1 infusion intrathecally [37]. The first patient developed side effects of meningoradiculitis with transient improvement to lower limb strength, and the second patient was pre-treated with immunosuppression and did not develop any side effects. Although lower levels of SOD1 were found on autopsy in the first patient, there was no reduction in CSF SOD1 in either patient at two weeks [37]. Patient 2 had also stable scores on a composite measure of ALS function and a stable vital capacity during a 12-month period [37].

### 3.3. Neurotrophins

Neurotrophins are signaling molecules that regulate neuronal function and can determine rates of apoptosis and modulate neuronal survival [43]. Most studies on neurotrophins in ALS have focused on insulin growth factor (IGF) and vascular endothelial growth factor (VEGF). When scAAV9-encoding IGF-1 was injected into SOD1 mice, it showed a marked reduction in motor neuron deterioration in the anterior horns of the spinal cord and delayed disease progression and onset [44]. In another study, injection of AAV9 expressing IGF-2 into SOD1-G93A mice showed a 10% increase in lifespan and therefore may be a protective factor for neuronal survival [45]. Lastly, scAAV9-VEGF-165 injection to SOD1-G93A mice showed improvements to prolong survival and motor strength [46]. Interestingly, when IGF-1 and VEGF were administered simultaneously, they did not show additive benefits, suggesting that these molecules may be acting on similar pathways [47].

### 3.4. CRISPR

CRISPR/Cas, which stands for “clustered regularly interspaced short palindromic repeats and CRISPR-associated protein”, was originally studied in bacteria [48] and is beginning to emerge in neurodegenerative diseases research. This strategy focuses on the correction of the mutant DNA in order to eliminate abnormal downstream pathways; thus, it could be theoretically considered as a one-time intervention. Currently, limited studies have been conducted in the ALS field. A 2017 study looked at CRISPR targeting the SOD1 gene where a modified AAV9 delivered *Staphylococcus aureus*-derived Cas9 (SaCas9) and a single-guide RNA (sgRNA) targeting the *SOD1* gene via the facial vein to neonatal SOD1G93A mice. There was evidence of decreased SOD1 expression in the spinal cords of these transgenic mice, with increase in motor neurons, delayed onset of disease and increased survival [49]. This study was followed by two studies, in 2020, which showed similarly decreased expression of SOD1 in the spinal cord and increased survivability [50,51]. The main limitation of these studies is the fact that treatment was administered to mice at a young age prior to exhibiting symptoms of ALS. It is therefore unclear how effective the treatment would be in older mice that would have started to exhibit symptoms related to ALS, since ALS diagnosis made by the revised El Escorial criteria requires symptoms in at least one anatomical region [52].

## 4. *C9orf72*

To date, *C9orf72* is the most significant gene discovery for ALS [15,16,17]; a mutation on chromosome 9 open reading frame 72, leads to an expansion of GGGGCC (G_4_C_2_) hexanucleotide repeats [15,16,17]. Accounting for up to 35–45% familial ALS [53], this prolific gene in ALS causes expansion to the repeat sequence located in the first intron of the *C9orf72*. Consequently, this causes disruption to the promoter region of this gene, responsible for controlling downstream transcription. Multiple studies have hypothesized this can lead to either a gain of or loss of function and affect subsequent protein synthesis [54]. Excess *C9orf72* protein is thought to be important in ALS, as this can lead to toxic accumulation of RNA, dipeptide protein aggregation, cytoplasmic transport disruption and nucleolar dysfunction [54]. In the normal population, hexanucleotide repeats in *C9orf72* are seen in the order of 20–30 s and were considered non-pathogenic [55]; in ALS, these repeats are commonly seen in the magnitude of hundreds [15,16,17]. However, recent evidence suggests expansions from as little as 24 repeats have been thought to contribute towards pathogenesis [56]. The relationship between repeat expansion size and phenotype is still not well understood and may arise from the variability between somatic mosaicism [3]. The mean age at onset is 57 for *C9orf72* ALS patients, with a median survival of 30–37 months [57]. FTD is also more prevalent in *C9orf72* ALS with faster disease progression and worsening clinical cognitive and behavioral changes [58,59]. It remains unclear whether *C9orf72* ALS patients have higher incidence of bulbar [58,60,61], or limb onset [57], which may give us a clue and potential target towards pathogenesis. Although no cure exists for ALS, the discovery of *C9orf72* ALS/FTD has initiated progress in developing targeted therapeutics and in elucidating our understanding of this fatal neurodegenerative disease.

### 4.1. Targeting C9orf72 Repeat-Expanded RNA or DNA

*C9orf72* repeat expansion through toxic gain and loss of functions such as impaired clearance of dipeptide proteins and excitotoxicity from accumulation of glutamate receptors can lead to premature neuronal death [62,63]. Therefore, inhibiting DNA transcription or reducing excess mRNA are potentially promising targets in halting *C9orf72* ALS/FTD disease progression.

***ASOs.*** ASOs targeting *C9orf72* RNA can inhibit *C9orf72*-specific pathologies [64,65,66,67] (e.g., nucleocytoplasmic trafficking deficits [68] and TDP-43 aggregation [68]) and improve survival in *C9orf72*-induced pluripotent stem cell (iPSC)-derived neurons or fibroblasts [68]. They also improve neurodegeneration in *C9orf72 Drosophila melanogaster* [68] and decrease sense RNA foci and dipeptide repeat proteins (DPRs) in *C9orf72* mice models [66,69]. These therapeutic effects have been demonstrated in a non-human study with a single intraventricular dose in BAC (bacterial artificial chromosomes) transgenic (G_4_C_2_)_450_ mice showing sustained reduction in RNA-foci and DPRs, with reversal of behavioral deficits [69]. An important point to note is that only *C9orf72* variants 1 and 3 (which carry the repeat expansion mutation) were targeted by ASOs without affecting variant 2 expression [69]; therefore, *C9orf72* abundance post treatment remained fairly similar between transgenic and wild-type animals. Furthermore, another study showed proof of concept in a single human, where intrathecal Afinersen (ASO5-2) was effective at safely suppressing C9orf72 transcripts and had an 80% reduction in poly(GP) dipeptide levels with functional stability in this individual over an 18 month period [70]. Notably, a phase I clinical trial of ASOs targeting *C9orf72* variants 1 and 3 (BIIB078) was recently completed by Ionis Pharmaceutical and Biogen Inc., in January 2022, and although it was well tolerated, it did not show any clinical benefit (ClinicalTrials.gov Identifier: NCT03626012) [71].

***RNA interference (RNAi).*** In one study, it was demonstrated that siRNA robustly decreased *C9orf72* mRNA in patients’ fibroblasts, but it did not affect nuclear RNA foci [65]. However, another investigation indicated that single-strand silencing RNAs decreased both sense and antisense RNA foci through reduction in mutants RNA transcript via RNAi and blockage of RBP binding to RNAs [72]. Hu et al. (2015) showed that engineered duplex RNAs enabled identification of difficult C/G targets and ultimately inhibited both sense and antisense RNA foci [73]. Although administration of synthetic siRNAs and ASOs is promising, they require repeated administration since they are used up when binding to excess mRNA, requiring multiple clinic visits and potentially creating a burden on the patient and their caregivers. Some studies have reported that AAV vector-delivered siRNAs derived from shRNA or miRNA scaffolds provided a longer-lasting therapeutic effect in other neurogenetic disorders such as Huntington’s disease [74,75]. Using this strategy, more recent studies have found that single administration of AAV5-delivered artificial miRNAs silenced *C9orf72* and decreased both nuclear and cytoplasmic RNA foci in both iPSC-derived motor neurons and ALS mouse model [76,77].

***Small compounds or genetic modifications targeting repeat RNA secondary structures***. The other approach is to utilize small compounds that can target the secondary structures of repeated (G_4_C_2_) RNAs (e.g., G-quadraplex, hairpin and R-loop structures) [68,78,79,80,81,82]. These small molecules can bind with the RNA secondary structures to prevent RAN translation as well as prevent sequestration of RBPs. One such molecule is the cationic porphyrin (5,10,15,20-tetra(N-methyl-4-pyridyl) porphyrin), also called TMPyP4, which can bind some G-quadruplex-forming sequences to distort the G-quadruplex formed by r(G_4_C_2_)_8_ and ablate the sequestration of RBPs [79]. It can also rescue nucleocytoplasmic transport defects and neurodegeneration in (G_4_C_2_)_30_ *Drosophila* [68]. Recent studies have also shown that these small molecules can bind to repeat RNA hairpin structures and significantly reduce RNA foci formation and poly-GP accumulation in (G_4_C_2_)_66_-cultured cells as well as iPSC-derived motor neurons from *C9orf72* ALS patients [82]. More detailed investigations are clearly needed to see whether these small compounds can also affect production of more toxic DPRs (i.e., arginine-rich dipeptides poly-PR and poly-GR) and exert therapeutic effects in vivo. Additionally, genetic modifications such as overexpression of *SETX* gene (encoding the RNA/DNA helicase senataxin) have been found to reduce levels of DNA double-stranded breaks through resolution of R-loops [83,84]. Notably, autosomal dominant mutations in the *SETX* gene are linked to a juvenile form of ALS [85]. *SETX* overexpression was also shown to reduce cellular toxicity in *C9orf72* expansion-expressing cells [86].

***Targeting repeat RNA transcription.*** Reducing (G_4_C_2_)_n_-containing RNA transcription could be considered as another therapeutic strategy in *C9orf72* ALS. Spt4 (the mammalian ortholog of Spt4 is Supt4h) and Spt5 are highly conserved transcription elongation factors that control RNA polymerase II processivity [87,88]. The therapeutic effects of Spt4 or Supt4h inhibition in reducing the transcription of CAG repeats in Huntington’s disease [89], has raised the possibility that inhibiting the Spt4 or Supt4h may be beneficial in other diseases with repeat expansion mutations. An interesting study by Kramer et al. (2016) demonstrated that *Spt4* deletion in the yeast *Saccharomyces cerevisiae* expressing *C9orf72* repeats led to a significant reduction in expression of (G_4_C_2_)_66_ or (C_4_G_2_)_66_ transcripts, as well as RNA foci and poly-GP levels [90]. Additionally, knockdown of endogenous *Spt4* with RNAi in (G_4_C_2_)_66_ *Caenorhabditis elegans* decreased both (G_4_C_2_)_66_ RNA and poly-GP levels, and also improved the survival of these worms [90]. Furthermore, *Spt4* RNAi partially suppressed the degenerative phenotype of the external and internal eye and improved the survival in (G_4_C_2_)_49_ *Drosophila*, and it almost completely suppressed the retinal thinning normally observed in (G_4_C_2_)_29_ *Drosophila* [90]. In the next step, Kramer et al. (2016) [90] treated cultured fibroblasts from three *C9orf72* ALS patients with siRNAs against *Supt4h1* or *Supt5h* (*siSupt4h1*, *siSupt5h*, respectively), decreasing both *Supt4h1* and *Supt5h* mRNA and protein levels, which led to a significantly reduced levels of *C9orf72* variant 3 mRNA, poly-glycine-proline DPRs, as well as both sense and antisense repeat RNA foci in *C9orf72* fibroblasts, without evidence of toxicity. On the other hand, treatment of *C9orf72* fibroblasts with an ASO targeting the *C9orf72* sense transcript exerted similar results with the key exception that foci formed of antisense (C_4_G_2_)-containing transcripts remained unaffected. Therefore, reducing the abundance of a single gene product, *Supt4h1* or *Supt5h*, decreased all three of the pathological characteristics of *C9orf72* ALS/FTD: sense RNA foci, antisense RNA foci, and DPRs. Notably, *Supt4h1* and *Supt5h* mRNA expression levels were positively correlated with levels of *C9orf72* variant 3 mRNA or poly-GP DPRs in the cerebellum of *C9orf72* ALS/FTD patients [90]. More recent investigation also revealed that a transcriptional regulator of RNA polymerase II, the CDC73/PAF1 complex (PAF1C), and its components *Leo1* and *Paf1*, are upregulated in transgenic (C_4_G_2_)_49_ *Drosophila*, (C_4_G_2_)_149_ mice, iPS cells from *C9orf72* ALS patients and frontal cortex from *C9orf72* ALS/FTD or *C9orf72* FTD cases [91]. Using RNAi to downregulate *PAF1C* components also selectively suppressed (G_4_C_2_)_49_ toxicity in multiple fly tissues, accompanied by a robust decrease in RNA and poly-GR DPRs production, and reduced both sense (G_4_C_2_)_66_ and antisense (C_4_G_2_)_66_ RNA in the yeast model [91]. Depletion of *Paf1* and *Leo1* in the fly nervous system selectively reduced the expression of long, toxic (G_4_C_2_)_49_ repeats [91]. The above studies have provided an intriguing insight into a novel approach for treatment of *C9orf72* ALS/FTD through suppression of specific transcriptional regulators (i.e., *PAF1C*, *Supt4h1* or *Supt5h*). However, before this approach can be executed in clinical trials, more studies are clearly needed to carefully explore the possible detrimental outcomes of global RNA processing besides *C9orf72* repeat expansions.

***Genome editing with CRISPR/Cas*.** In a study by Gaj et al. (2017), AAV9 vectors containing CRISPR/Cas9 were administered into the facial veins of one-day-old transgenic *G93A-SOD1* ALS mice to disrupt mutant *SOD1* expression [49]. They found exciting results showing a >2.5-fold drop in mutant-SOD1 protein levels in the thoracolumbar spine, 50% more motor neurons at the end stage, 37% delay in ALS onset, and 25% increased survival [49]. In the same year, two separate studies also reported utility of CRISPR/Cas9 in targeting either (G_4_C_2_) repeat DNA [92] or (G_4_C_2_) repeat RNA [93] in order to reduce repeat RNA transcription or levels of RNA foci/DPRs, respectively. One important issue that needs to be considered while interpreting these data is that the treatment was given to the transgenic mice from birth before they exhibited any ALS phenotype (which typically takes 90 days after birth in these transgenic mice); thus, it is still unclear whether this approach would provide an equal outcome in older mice when the disease is active. Clinically diagnosing ALS is an arduous task, taking an average of 11.5 months, due to delays from when patients first elicit symptom onset to presentation, and requiring additional testing to rule out other diseases [94]. As a result, treatments that may work early on in disease progression may become less efficacious with any diagnostic delay. So far, there is no consensus on recommendation for genetic screening of asymptomatic family members of affected ALS patients or the general population, and therefore, potential preventive treatments that require administration before symptom onset are harder to conduct clinical trials on. Therefore, as genetic testing becomes more economical, genetic targets can be expanded in future clinical trials. These results, although limited, have opened a new avenue in translational research aiming to target abnormal DNA/RNA in *C9orf72* ALS/FTD using CRISPR/Cas9. Important concerns including ethical issues, safe drug delivery methods, and potential adverse outcomes need to be addressed before this approach takes its application in clinical practice.

### 4.2. Targeting DPRs

Although strategies to target *C9orf72* repeat expansion DNA or RNA as upstream pathologic pathways are promising and may help correct related downstream pathways such as DPR toxicity or nucleocytoplasmic trafficking deficits, other therapeutic approaches that target downstream pathways directly (such as DPRs) are currently being investigated. To date, three approaches have been proposed to directly reduce the pathologic aspects of DPRs in *C9orf72* ALS/FTD.

***Antibody immunization against DPRs and their cell-to-cell transmission.*** This strategy is similar to other neurodegenerative diseases in which abnormal proteins such as amyloid-β, tau, and α-synuclein [95,96] are targeted by antibodies. In a study by Zhou et al. (2017), treatment of either (GA)_175_-GFP-transfected HEK293 cells or rat primary neurons with anti-GA antibody reduced (GA)_175_-GFP aggregation in both cell cultures compared to isotype controls [97]. Furthermore, pre-incubation with anti-GA antibody inhibited (GA)_80_ uptake from *C9orf72* brain extracts into HEK293 cells, providing evidence for inhibitory effects of these antibodies on the seeding activity of brain extracts from *C9orf72* ALS cases [97].

***Removal/clearance of toxic DPRs.*** This approach has been tested in a recent study, where it was shown that overexpression of the small heat shock protein B8 (HSPB8) facilitated the autophagic removal of all five DPRs in immortalized motoneurons cell lines NSC34 expressing each single DPR [98]. Another potential target, a protein kinase A (PKA) inhibitor H89, has been shown to reduce DPK levels in patient-derived iPSC motor neurons [99]. Another study [100] also reported that an anticoagulation-deficient form of activated protein C, called 3K3A-APC, rescued neuronal defects in both *C9orf72* and sporadic ALS-induced motor neurons (iMNs), through rescuing the defective autophagosome, thereby reducing *C9orf72* DPR levels, restoring nuclear TDP-43 localization, and improving the survival of both *C9orf72* and sporadic ALS iMNs [100].

***Inhibiting DPR production.*** Through performing genome-wide CRISPR/Cas9 screens for modifiers of DPR protein production in human cells, it was recently found that DDX3X (DEAD-Box Helicase 3 X-Linked), an RNA helicase, suppressed the repeat-associated non-AUG translation of G_4_C_2_ repeats through direct binding to repeat RNAs [101]. Increasing the expression of *DDX3X* led to a decrease in DPR levels, rescued nucleocytoplasmic transport defects, and improved survival of iPSC-differentiated neurons from ALS cases [101]. Compounds that inhibit 10hosphor-eIF2α signaling (i.e., ISRIB (integrated stress response inhibitor) and GSK2606414) have been also shown to suppress RAN translation, thereby inhibiting DPR production and preventing related cellular toxicity [102].

### 4.3. Targeting Nucleocytoplasmic Transport System

Targeting nuclear transport is another approach being investigated in recent years, which includes three main strategies:

***Genetic modification.*** Reducing the expression of exportin using an RNAi strategy or overexpressing importin α, two important proteins involved in active transport of large (>40 kD) proteins, in (G_4_C_2_)_30_ *Drosophila* demonstrated rescue of neurodegeneration in flies’ eyes [68]. A separate study also found that knockdown of the RBP SRSF1, which acts as a nuclear export adaptor protein triggering RNA nuclear export, using an RNAi strategy in (G_4_C_2_)_36_ *Drosophila* restored motor function, reduced the production of both sense and antisense poly-GP DPRs, and mitigated astrocyte-mediated neurotoxicity in these (G_4_C_2_)_36_ flies [103]. *SRSF1* knockdown in iPSC-derived motor neurons from *C9orf72* ALS patients provided a neuroprotective effect against neuronal cell death [103]. This data suggests that knockdown of certain nuclear export proteins (i.e., exportin and SRSF1) can potentially prevent export of toxic RNA repeats to the cytoplasm, thereby inhibiting the downstream pathway related to their toxicity (e.g., production of DPRs).

***Indirect effect using ASOs targeting Ataxin-2.*** An interesting study by Zhang et al. (2018) demonstrated that *Ataxin-2* may contribute to the nucleocytoplasmic defects in *C9orf72* ALS/FTD through disrupting the stress granule assembly [104]. Knockdown of *Ataxin-2* expression using ASOs suppressed nucleocytoplasmic transport defects, TDP-43 pathology, and neurodegeneration in both (G_4_C_2_)_30_ *Drosophila* and iPS motor neurons derived from *C9orf72* ALS patients [104]. Similar results were obtained when stress granule inhibitors were used [104].

## 5. TARDBP (TDP-43)

TDP-43 is a DNA/RNA binding protein, encoded by *TARDBP* gene. In 2006, TDP-43 was discovered as a major component of the pathological cytoplasmic inclusions in ALS and FTD [105,106]. Subsequently, mutations in *TARDBP* gene were discovered as causative factors in ALS [18,107,108,109]. So far, more than 50 mutations in *TARDBP* gene have been identified [110]. While mutations in *TARDBP* gene cause up to 5% of familial ALS and 1% of sporadic ALS, TDP-43 protein is found in the cytoplasmic aggregates of most ALS and FTD cases [111,112,113,114]. TDP-43 is known to regulate RNA processing including RNA splicing, mRNA transport, translation as well as non-coding RNA regulation [115,116]. It is normally localized in the nucleus, but it contains both nuclear localization and export signals, which can bring itself back and forth between the nucleus and the cytoplasm [117].

The cytoplasmic aggregates of TDP-43 are thought to be related to a loss of TDP-43 function in the nucleus, and a gain of toxic TDP-43 function in the cytoplasm, or both. Studies using a variety of *TARDBP* knock out/down as well as overexpression animal models showed both loss of TDP-43 and overexpression of TDP-3 as causative elements for ALS [118,119,120,121], which highlights the importance of tight regulation of TDP-43. In addition, several post-translational modifications including ubiquitination, phosphorylation, and proteolytic cleavage were found to be associated with pathological TDP-43 [18,122,123].

### 5.1. Targeting TARDBP

The level of TDP-43 and its localization in the cell need to be tightly regulated. Therefore, gene therapy controlling TDP-43 expression and/or localization could be a good treatment option for patients with *TARDBP* ALS/FTD.

***ASOs***. Currently, there is no clinical development of ASOs directly targeting *TARDBP.* However, ASOs targeting *Ataxin-2* were tested in TDP-43 animal models, which reduced TDP-43 aggregation and pathology [124]. Additionally, ASO targeting knockdown of CHMP 7 improved neuronal survival in iPSC-derived spinal neurons and postmortem human tissue [125].

***RNAi***. iPSCs derived from ALS patients with known genetic mutations can be used for testing gene therapies. A previous study showed a reduction in nuclear and cytoplasmic TDP-43 after administration of siRNA targeting M337V to TDP-43^M337V^-iPSCs [126]. Further studies will be needed, but this siRNA approach specifically targeting known *TDP-43* mutations might be a potential treatment option for familial ALS with *TDP-43* mutations.

***Genome editing with CRISPR/Cas***. TDP-43 regulates mRNA splicing including *Sort1* mRNA encoding Sortilin protein. It has a known role in regulating brain-derived neurotrophic factor (BDNF), which is essential for synaptic plasticity, neuronal survival as well as differentiation [127]. Tann et al. (2019) successfully corrected M337V mutation in TDP-43^M337V^-iPSCs using CRISPR/Cas9 and proved that M337V mutation impairs BDNF secretion and synaptic plasticity through altering Sortilin splicing [128]. Gene editing using CRISPR/Cas9 has opened a new avenue of therapeutic approach for ALS. However, a considerable amount of future work will be needed to optimize this therapy for this to be applied to clinical practice.

### 5.2. Targeting Nucleocytoplasmic Transport System

TDP-43 has a nuclear export signal. Since the cytoplasmic accumulation of TDP-43 associated with a loss of TDP-43 function in the nucleus and gain of toxic TDP-43 function in the cytoplasm are thought to be causative of ALS, new compounds that selectively inhibit nuclear export were developed [129]. These compounds showed modest improvement in motor neuronal survival and partial rescue of motor phenotype in the TDP-43 overexpressing animal model. However, they failed to show a reduction in nuclear export of TDP-43 [129].

## 6. *FUS*

In 2009, mutations in *FUS* gene in chromosome 16 were discovered as a causative factor for ALS [19,20]. More than 50 different mutations in *FUS* gene have been identified, and these mutations cause up to 4% of familial and 1% of sporadic ALS [130,131] and, more specifically, juvenile onset ALS [132,133]. LMN signs with a younger age onset and aggressive disease course are predominant, with bulbar and spinal onset being the types that are more frequent in *FUS* ALS cases (77, 100). However, cognitive symptoms and FTD are rare with mutations in *FUS* [110,134,135].

FUS is mainly localized in the nucleus. Although precise physiological function of FUS is not well understood, it is known to regulate RNA splicing, mRNA trafficking, and DNA repair [130,136]. In addition, FUS plays a role in paraspeckle formation, which provides cellular defense against different types of cellular stress [137].

Most described mutations in *FUS* are missense mutations, clustered within the 3′ arginine/glycine-rich region and a nucleus localization signal domain. These mutations mainly cause cytoplasmic mislocalization of FUS, which leads to FUS-immunoreactive inclusions attributed to neuronal degeneration in ALS [138]. It is thought that both loss of function in the nucleus and gain of toxic function in the cytoplasm of FUS play a role in pathogenesis of *FUS* ALS [139,140].

### Targeting FUS

Since *FUS* mutations-related ALS is rare, gene therapy approaches targeting *FUS* are not as well incentivized as other more common genes such as *SOD1* and *C9orf72*.

***ASOs.*** A recent multi-center, phase 1–3 study of ASOs targeting *FUS* gene, called Jacifusen, has been initiated by the Eleanor and Lou Gehrig ALS Center at Columbia University Irving Medical Center, supported by ALS association and Project ALS, and it represents the first clinical trials targeting *FUS* (Table 1).

***Genome editing with CRISPR/Cas***. Additional studies have used CRISPR/Cas9 to study *FUS* pathogenesis using iPSCs derived from ALS patients with *FUS* mutations [141,142,143,144,145]. The first CRISPR/Cas-9-mediated *FUS* G1566A correction was demonstrated by Wang et al. [141]. After this study, CRISPR/Cas-9-mediated correction of *FUS* H517Q mutation showed that the abnormal activation of mitogen-activated protein kinase (MAPK) signaling is related to *FUS* mutation-mediated neurodegenerative process in ALS [142]. Another study with CRISPR/Cas9-mediated *FUS* R521H correction proved that pathological phenotypes observed in the motor neurons with *FUS* mutation could be rescued by gene correction [145]. In addition, correction of *FUS* P525L and R521H mutations using CRISPR/Cas9 was able to rescue DNA ligation defects which were decreased in *FUS* ALS patient-derived motor neurons [143]. Even though further studies are required for CRISPR/Cas9 mediated gene editing to be applied in clinical practice, it may facilitate the development of novel therapies in ALS.

## 7. Conclusions and Perspectives

Up to 10% of ALS cases is gene related, meaning that there are many discrete targets for molecular therapies. There are several cellular and animal models specific to ALS-related genes with newer models continuing to be developed, which has enabled us to improve our mechanistic understanding of the disease and allowed us to explore new genetic targets, exciting treatments, and novel vectors for directed administration of molecular therapies. Some gene therapies have shown significant effects in animal and cell models at improving functional outcomes of disease and some have also shown effectiveness in small group human studies, clearing the pathway for larger clinical trials.

Since ALS is only one of multiple gene-related neurodegenerative diseases, a comparison to other similar diseases such as spinal muscular atrophy shows that genetic treatments in ALS is possible. In SMA type I and II, a childhood neurodegenerative disorder, an ASO, nusinersin (Spinraza), and gene therapy AVXS-101 (zolgensma) via AAV9 vector, are now gold-standard therapies, with zolgensma being effective at a single intravenous dose, if treated before the age of two, affording a normal life for these patients. However, in ALS, it is less likely that a single dose therapy would be sufficient in stopping the disease, since ASOs would not affect new transcription of RNA, and target diseased neurons via gene therapy would be difficult, due to the number of neurons already being affected. Furthermore, intravenous and intrathecal administration would be challenging, since for current ALS medications which require IV administration, such as edaravone (Radicava), patients are required to either have multiple visits to infusion centers or, if their insurance permits, have home therapy via port-a-cath. As the ALS disease burden progresses, patients may face difficulties in transportation and time to reach infusion sites, decreasing their overall quality of life.

In addition, the multiple genes involved in ALS further complicates potential treatments, since treatments are required to model the individualized genetic makeup. This has become more feasible with improvements to genome sequencing with the technology being streamlined, and now it is affordable to order online personalized DNA reports and enter online genetic ancestry databases. With this, there is potential for future clinic visits to include standardized ALS gene panels, or it could even be offered to family members who may be carriers of the disease, which is currently mainly limited to clinical trials or those who can pay out of pocket. Because of this inaccessibility, clinical trials describing management of ALS-related gene carriers is limited. There is only one ongoing clinical trial (ATLAS trial) that is utilizing the second-generation ASO tofersen (or BIIB067) (which targets *SOD1*) in carriers of *SOD1* variants associated with high or complete penetrance and rapid disease progression who do not yet have clinical manifestations of disease but have elevated neurofilament levels (ClinicalTrials.gov Identifier: NCT04856982) [146]. The idea came from the fact that in a subset of participants in the Pre-Symptomatic Familial ALS study (a longitudinal natural history/biomarker study of asymptomatic people at high genetic risk for ALS [ClinicalTrials.gov Identifier: NCT00317616] since 2007) [147], it was found that in subjects with a *SOD1* variant associated with rapid disease progression (e.g., p.Ala5Val [A5V; A4V]) and in phenoconverters (at-risk persons observed both before and after the emergence of clinically manifest disease) during follow-up, elevated serum neurofilament levels (most notably neurofilament light chain, NfL) were observed 6–12 months before the phenoconversion [148,149]. With these observations and the recent potential beneficial effects of tofersen in the reduction of total CSF SOD1 protein and plasma NfL in symptomatic patients with SOD1-ALS [35], the results of the ATLAS trial will inform us as to whether initiation of tofersen can delay the onset or slow the progression of ALS in this high-risk population of presymptomatic *SOD1* carriers [146]. This also emphasizes the fact that better screening of the disease could lead to wider clinical trials to identify and include pre-symptomatic patients and carriers with pre-disposition to ALS, improve understanding of disease progression and potentially develop preventative treatments.

Given the fact that pre-symptomatic ALS subjects (irrespective of being a carrier of a pathogenic gene variant or not) have no or subtle clinical symptoms, biomarkers may play an essential role in evaluating this stage of disease. Although there has been a great advancement in this field (e.g., studies on neurofilaments as promising biomarkers), finding a unique and reliable biomarker is still a challenge, which is mainly due to the ALS disease heterogeneity and variability in disease onset/course. Nevertheless, assessment and longitudinal monitoring of potential biomarkers in pre-symptomatic patients seems critical as they may serve to characterize the time-line for disease onset prior to the clinical manifestation and to serve as critical predictors of disease progression.

Avenues are being explored for gene therapy in ALS, cellular and animal models with *C9orf72*, *SOD1*, *TARDP-43* and *FUS* mutations allowing for study of the disease in a replicated human model of disease. Advances in these models have improved understanding of pathogenic mechanisms (Figure 1) and established the foundation for ALS clinical trials in humans (Table 1). Tofersen (BIIB067) ASO for SOD1 gene was shown in a phase 1 study to be safely tolerated and was found to lead to a reduction in CSF SOD1 protein in fast ALS progressors. Although initial phase 3 clinical trials on fast ALS progressors did not show a statistically significant decline in CSF SOD1, a longer follow-up study currently in process may indeed show us the effect of treatment in both fast- and slow-progressing ALS patients in a 7-year follow-up period, since this timeframe would encapsulate the disease course of most ALS patients. Interestingly, human studies with intrathecal administration of AAV-miR-*SOD1* found transient improvements to muscle strength, and therefore, repeated studies with a higher number of patients and perhaps utilizing different doses, if tolerated, may substantially change the future management of *SOD1* ALS. These are some of the many ongoing clinical trials in ALS, and combined with improved genetic testing accessibility, better animal models and the ever-expanding framework of treatments, a renewed vigor in the search for genetic solutions to ALS is established.

## Figures and Tables

**Table 1:** Gene Therapy Clinical Trials in Amyotrophic Lateral Sclerosis.

Agent	Mechanism of Action	Primary Measure Outcomes	Trial Design	N	Sites of Study	Status	CTI	Primary Outcome
BIIB067 or Tofersen (VALOR Trial)	ASO against *SOD1* mRNA	Safety, tolerability, pharmacokinetics, biomarkers, ALSFRS-R change at 28 weeks	Phase 3, randomized, quadruple-blinded, placebo-controlled	183	USA, Canada, Europe	Complete	NCT-02623699	N/A
		AE and SAE up to 248 weeks	Extension of Phase 3, placebo-cotrolled, open label	183	USA, Canada, Europe	Active	NCT-03070119	N/A
ISIS 333611 [33,34]	ASO against *SOD1* mRNA	Safety, tolerability, and pharmacokinetics at unknown time	Phase 1, quadruple-blinded, randomized, placebo-controlled	33	USA	Complete	NCT-01041222	No AE, Well tolerated, dose-dependent CSF and plasma concentrations
[37]	AAV-miR-*SOD1*	Safety, tolerability, and pharmacokinetics	Open-label	2	USA	Complete	N/A	Meningoradiculitis in case 1, but not in case 2 with immunosuppressive therapy; Transient improvement in muscle sctregnth in case 1;
BIIB078	ASO against *C9orf72* mRNA	Safety at 323 days	Phase 1, quadruple-blinded, randomized, placebo-controlled	90	USA, Canada, Europe	Complete	NCT-03626012	N/A
SB-509 [38]	Plasmid encoding a zinc finger DNA-binding protein transcription factor (ZFP TF(TM)) designed to up-regulate the expression of the gene encoding vascular endothelial growth factor (VEGF-A)	Change in ALSFRS-R at 11 months	Phase 2, open label	45	USA	Complete	NCT-00748501	Safe, delayed deterioration in ankle and toe strength in 40% of treated subjects
ION363 (Jacifusen)	ASO against *FUS* mRNA	Change in ALSFRS-R and Ventilation Assistance-free survival (VAFS) at 505 days	Phase 1–3, double-blinded, randomized, placebo-controlled	77	USA, Canada, Belgium, UK	Active	NCT-04768972	N/A

## Data Availability

Not applicable.

## Abbreviations

Abbreviation	Definition
*AAV*	Adeno-Associated Virus
*AAV*-*miR*-*SOD1*	Adeno-Associated Virus Micro-Ribonucleic Acid against Cu/Zn Superoxide Dismutase
*AE*	Adverse Events
*ALS*	Amyotrophic Lateral Sclerosis
*ALSFRS-R*	Revised Amyotrophic Lateral Sclerosis Functional Rating Scale
*ASO*	Antisense Oligonucleotides
*BAC*	Bacterial Artificial Chromosomes
*BDNF*	Brain-Derived Neurotrophic Factor
*Cas9*	CRISPR-Associated Protein 9
*cDNA*	Complementary Deoxyribonucleic Acid
*CHMP7*	Charged Multivesicular Body Protein 7
*CNS*	Central Nervous System
*CRISPR*	Clustered Regularly Interspaced Short Palindromic Repeats
*CSF*	Cerebrospinal Fluid
*C9ORF72*	Chromosome 9 Open Reading Frame 72
*DMD*	Duchenne Muscular Dystrophy
*DNA*	Deoxyribonucleic Acid
*DPK*	Dependent Protein Kinase
*DPR*	Dipeptide Protein Repeats
*fALS*	Familial Amyotrophic Lateral Sclerosis
*FTD*	Frontotemporal Dementia
*FUS*	Fused in Sarcoma
*HSPB8*	Small Heat Shock Protein B8
*IGF*	Insulin Growth Factor
*IMN*	Induced Motor Neurons
*iPS*	Induced Pluripotent Stem Cells
*isRIB*	Integrated Stress Response Inhibitor
*LMN*	Lower Motor Neuron
*MAPK*	Mitogen-Activated Protein Kinase
*miRNA*	Micro Ribonucleic Acid
*mRNA*	Messenger Ribonucleic Acid
*PLS*	Primary Lateral Sclerosis
*PMA*	Progressive Muscular Atrophy
*RAN*	Repeat Association non-AUG
*RBP*	Ribonucleic Acid Binding Protein
*RISC*	Ribonucleic Acid-Induced Silencing Complex
*RNA*	Ribonucleic Acid
*RNAi*	Ribonucleic Acid Interference
*saCas9*	Staphylococcus Aureus CRISPR-associated protein 9
*SAE*	Severe Adverse Events
*sALS*	Sporadic Amyotrophic Lateral Sclerosis
*sgRNA*	Single Guide Ribonucleic Acid
*shRNA*	Short Hairpin Ribonucleic Acid
*siRNA*	Small Interfering Ribonucleic Acid
*SMA*	Spinal Muscular Atrophy
*SOD1*	Cu/Zn Superoxide Dismutase
*SRSF1*	Serine/Arginine-rich Splicing Factor 1
*TARDBP 43*	Transactive Response DNA-Binding Protein 43
*UMN*	Upper Motor Neuron
*VAF*	Ventilation Assistance-Free Survival
*ZFP TF*	Zinc Finger Protein Transcription Factor
